# The Level of Anti-Viral Antigen-Specific Antibodies to EBNA-1 in the Serum of MS Patients Does not Depend on the Severity of the Disease

**DOI:** 10.1134/S1607672924700753

**Published:** 2024-03-12

**Authors:** L. A. Ovchinnikova, S. S. Dzhelad, T. O. Simaniv, M. N. Zakharova, A. G. Gabibov, Y. A. Lomakin

**Affiliations:** 1 Shemyakin–Ovchinnikov Institute of Bioorganic Chemistry, Russian Academy of Sciences, Moscow, Russia; 2 Research Center of Neurology, Moscow, Russia; 3 Moscow State University, Moscow, Russia

**Keywords:** MS, multiple sclerosis, EBNA-1, autoantibodies, EBV, Epstein–Barr virus

## Abstract

Multiple sclerosis (MS) is an autoimmune neurodegenerative disease leading to inevitable disability and primarily affecting the young and middle-aged population. Recent studies have shown a direct correlation between the risk of MS development and Epstein–Barr virus (EBV) infection. Analysis of the titer of EBV-specific antibodies among patients with MS and healthy donors among Russian population confirmed that MS is characterized by an increased level of serum IgG binding EBNA-1 (EBV nuclear antigen 1). The number of patients with elevated levels of EBNA-1-specific antibodies does not differ statistically significantly between two groups with diametrically opposite courses of MS: benign MS or highly active MS. It can be assumed that the primary link between EBV and the development of MS is restricted to the initiation of the disease and does not impact its severity.

Multiple sclerosis (MS) is a chronic immune-mediated demyelinating disease of the central nervous system (CNS). MS is an extremely heterogeneous disease, the development of which can be triggered by a combination of factors such as genetic predisposition, bacterial and viral infections (primarily herpes viruses), unfavorable environment, and bad habits [1]. However, the mechanism of initiation of this disease has not been fully established. In the last decade, a special role in the development of this disease has been assigned to the Epstein–Barr virus (EBV) [2, 3]. In the case of EBV infection, the risk of MS development increases 32 times [2]. It should be noted that antiviral antibodies are detected in the blood on average 10 years before the first clinical manifestations of MS [4]. The virus infects human B cells and then enters a latent phase, during which the expression of viral proteins markedly decreases compared to active infection [5]. This process partially contributes to the persistence of infected B cells and the maintenance of infection in the human body. However, the most important proteins for maintaining the activity of EBV, among which the most studied is nuclear antigen-1 (EBNA-1), which is required for maintaining the viral genome, continue to be expressed even in the latent phase [6].

Previously, the presence of cross-reactive monoclonal antibodies that simultaneously recognize a fragment of the viral antigen EBNA-1 (386–405 aa) and the autoantigen of glial cells GlialCAM (370–389 aa) was shown in patients with MS [7]. The study of the titer of antibodies to viral antigens in patients with MS will help understand how the level of activation of the immune system in response to EBV is associated with the severity of MS. Thus, the purpose of our study was to determine the level of antiviral antibodies to EBNA-1 (386–405 aa) in the blood of patients with aggressive MS (highly active MS (HAMS)) and a less severe form of the disease, benign MS (BMS) [8, 9].

## MATERIALS AND METHODS

The amount of antigen-specific antibodies to EBNA-1 (386–405 aa) in the blood sera of patients with MS and healthy donors (HD) was determined by enzyme-linked immunosorbent assay (ELISA). In this work, we consider two clinical variants of the course of relapsing–remitting MS—benign MS (BMS) and a more aggressive one, highly active MS (HAMS) [8, 9]. BMS is characterized by a slow progression in the absence of specific treatment and a relatively low Kurtzke Expanded Disability Status Scale (EDSS) score, less than 4. Patients with this form of MS often exhibit various cognitive impairments [10]. The more severe form of the disease, HAMS, is characterized by a high rate of formation of new lesions, extensive demyelination in the CNS and an incomplete recovery during remission and the presence of two or more relapses per year [11]. To compare MS with other neurological diseases, we measured levels of antiviral antigen-specific antibodies in patients with amyotrophic lateral sclerosis (ALS) and neuromyelitis optica spectrum disorders (NMOSD). These diseases are characterized by symptoms that partially overlap with MS, but their pathogenesis is significantly different. The level of antibody response to viral antigens was studied on samples of HD (*n* = 19) and MS (*n* = 29) (divided into BMS (*n* = 9), HAMS (*n* = 20), ALS (*n* = 14), and NMOSD (*n* = 5)) (Table 1).

**Table 1.** Characteristics of patients with MS, ALS, and NMOSD and healthy donors participating in the study

	HD	MS	BMS	HAMS	ALS	NMOSD
Quantity	19	29	9	20	14	5
Age (years)	42 ± 9	39 ± 11	48 ± 11	35 ± 8	56 ± 9	55 ± 18
Sex (% women)	52%	66%	75%	44%	64%	100%
Disease duration 1–5 years	–	51.7%	11.1%	70%	100%	60%
Disease duration 6–10 years	–	13.8%	11.1%	15%	–	20%
Disease duration more than 10 years	–	34.5%	77.8%	15%	–	20%
EDSS		3.7 ± 1.8	3.6 ± 1.7	3.8 ± 2.0	–	–

HD—healthy donors, MS—multiple sclerosis, BMS—benign MS, HAMS—highly active MS, EDSS—Expanded Disability Status Scale [12] (from 0 to 10, where 0 is normal, 10 is death from MS).

The level of antibody response was determined using a chemically synthesized EBNA-1 peptide (386–405 aa, SQSSSSGSPPRRPPPGRRPF) with a purity of >90%. This allows to avoid a false-positive signals resulting from the binding of by-products, which are often formed during prokaryotic protein expression, by nonspecific serum antibodies. The peptide was loaded on the bottom of the wells of a sorbing plate at a concentration of 1 μg/mL in carbonate buffer (pH 9.0), and the plate was incubated for 16 h at 4°C. Then, the remaining binding sites on the bottom of the wells were blocked by incubating with a solution of 2% skimmed milk powder in carbonate buffer (pH 9.0) for 1 h at 37°C with stirring. Blood serum was diluted 100 times with conjugate buffer (phosphate-buffered saline (pH 7.4), 0.05% Tween-20, and 0.5% skim milk powder). After incubation at room temperature for 1 h with stirring, the plates were washed 5 times with washing buffer (phosphate-buffered saline (pH 7.4) and 0.1% Tween-20). The bound antibodies were detected with secondary antibodies to the F(ab′)2 fragment of human IgG antibodies conjugated to horseradish peroxidase (Sigma-Aldrich, A2290). After incubation with the secondary antibodies, six washing cycles were performed, followed by the addition of 3,3',5,5'-tetramethylbenzidine hydrochloride (TMB), which is a substrate of horseradish peroxidase. The reaction was stopped by adding a solution of 10% sulfuric acid. The binding signal was recorded using a Varioskan LUX multimode microplate reader at a wavelength of 450 nm. The results were statistically processed using GraphPad Prism software version 9.

## RESULTS AND DISCUSSION

**Fig. 1.**
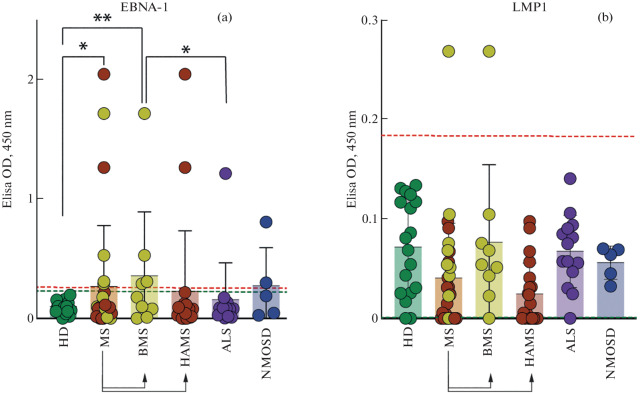
The level of antigen-specific IgG to EBV fragments in human serum. (a) Binding of chemically synthesized EBNA-1 peptide (386–405 aa). (b) Binding of chemically synthesized irrelevant peptide LMP1 (7–26 aa). Designations: HD—healthy donors, MS—patients with multiple sclerosis, BMS—patients with benign multiple sclerosis, HAMS—patients with highly active multiple sclerosis, ALS—patients with amyotrophic lateral sclerosis, NMOSD—patients with neuromyelitis optica spectrum disease. The red dotted line indicates the threshold optical density value above which the serum is considered seropositive for the test antigen. The dotted green line indicates the control signal of binding of the test antigen by IVIG (pooled IgG from 1000 healthy donors) (*p < 0.05; **p < 0.01, Pearson’s chi-square).

To verify the measurement of antibody titers specifically binding the cross-reactive viral peptide EBNA-1 (386–405 aa), potentially associated with the development of MS, and not the overall titer of antiviral antibodies, we performed an additional analysis of antigen-specific antibodies to the control fragment of another EBV latent phase protein, latent membrane protein 1 (LMP1). The inclusion of this control allows us to verify that the increased level of reactivity of antibodies to EBNA-1 (386–405 aa) in patients with MS is not associated with general immunoinflammation or the persistence of EBV in the body. The results of the study are presented in Fig. 1. The level of antiviral antibody response to EBNA-1 (386–405 aa) is significantly increased in patients with MS, regardless of the severity of the disease, as compared to HD. In addition, a statistically significant difference in the number of seropositive donors is observed in patients with BMS as compared to HD. Within the MS group, the level of binding of the viral antigen EBNA-1 (386–405 aa) and the number of seropositive donors are not statistically significantly different between patients with BMS and HAMS. In contrast to MS, none of the other diseases (ALS and NMOSD) showed a statistically significant difference in the level of antigen-specific antibodies to EBNA-1 (386–405 аа) compared to HD. It should be noted that the level of binding of LMP1 peptide by serum antibodies in patients with MS is comparable to that in the groups of healthy donors and patients with other neurological diseases. This level remains relatively low, confirming the association between MS and antigen-specific antibodies to EBNA-1 fragment (386–405 aa).

## CONCLUSIONS

**Fig. 2.**
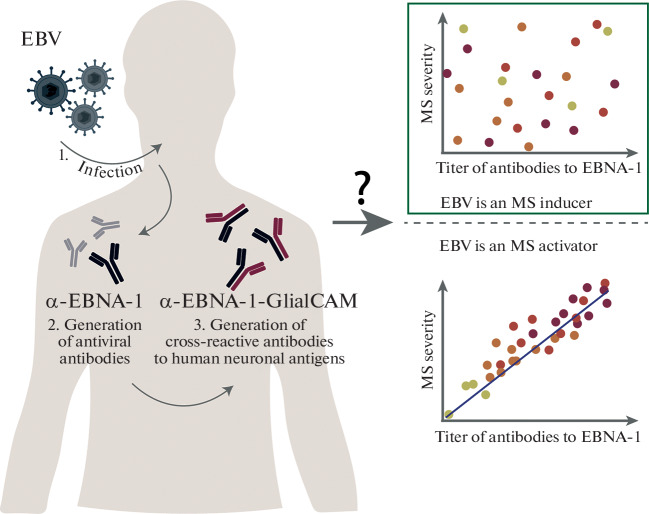
The proposed scheme of the involvement of the Epstein–Barr virus in the development of multiple sclerosis. (1) Epstein–Barr virus enters the human body and infects B cells. (2) Viral antigens accumulate (for example, EBNA-1, VCA, etc.), and the formation of antiviral antibodies to these antigens begins. (3) The resulting antiviral immunoglobulins become cross-reactive to human neuronal antigens during the process of maturation and somatic hypermutation. Designations: EBV—Epstein–Barr virus, MS—multiple sclerosis.

Based on the results of recent studies, it can almost certainly be concluded that EBV is an initiating factor of MS; however, its impact on the severity of MS has not been fully established (Fig. 2). One of the most likely elements of EBV inducing the formation of cross-reactive pathogenic antibodies in MS is the EBNA-1 antigen (386–405 aa). In this work, we compared the levels of binding of a synthetic peptide of the viral antigen EBNA-1 (386–405 aa) by the antibodies from the blood sera of patients with different forms of MS (BMS and HAMS). According to our data, the level of antiviral antibodies in serum does not depend on the severity of MS. Based on the obtained data, it can be assumed that EBV functions as an inducer rather than as an activator of MS (Fig. 2). However, the role of EBV in the MS progression is not completely clear. Possibly, further study of this influence and a more detailed analysis of patients with primary and secondary progressive types of MS will help adjust therapy to slow down the development of this disease.
